# Overexpression of microRNA-205-5p promotes cholangiocarcinoma growth by reducing expression of homeodomain-interacting protein kinase 3

**DOI:** 10.1038/s41598-023-49694-x

**Published:** 2023-12-17

**Authors:** Aye Myat Mon, Kitti Intuyod, Sirinapha Klungsaeng, Apinya Jusakul, Thatsanapong Pongking, Worachart Lert-itthiporn, Vor Luvira, Chawalit Pairojkul, Tullayakorn Plengsuriyakarn, Kesara Na-Bangchang, Somchai Pinlaor, Porntip Pinlaor

**Affiliations:** 1 Medical Technology Program, Faculty of Associated Medical Sciences, Khon Kaen University, Khon Kaen, 40002 Thailand; 2 Department of Pathology, Faculty of Medicine, Khon Kaen University, Khon Kaen, 40002 Thailand; 3 Department of Parasitology, Faculty of Medicine, Khon Kaen University, Khon Kaen, 40002 Thailand; 4 Centre for Research and Development of Medical Diagnostic Laboratories, Faculty of Associated Medical Sciences, Khon Kaen University, Khon Kaen, 40002 Thailand; 5 Biomedical Sciences Program, Graduate School, Khon Kaen University, Khon Kaen, 40002 Thailand; 6 Department of Biochemistry, Faculty of Medicine, Khon Kaen University, Khon Kaen, 40002 Thailand; 7 Department of Surgery, Faculty of Medicine, Khon Kaen University, Khon Kaen, 40002 Thailand; 8 Graduate Program in Bioclinical Sciences, Center of Excellence in Pharmacology and Molecular Biology of Malaria and Cholangiocarcinoma, Chulabhorn International College of Medicine, Thammasat University (Rangsit Campus), Pathum Thani, 12120 Thailand; 9 Cholangiocarcinoma Research Institute, Faculty of Medicine, Khon Kaen University, Khon Kaen, 40002, Thailand

**Keywords:** Cancer, Molecular biology, Oncology

## Abstract

The microRNA miR-205-5p has diverse effects in different malignancies, including cholangiocarcinoma (CCA), but its effects on CCA progression is unclear. Here we investigated the role and function of miR-205-5p in CCA. Three CCA cell lines and human serum samples were found to have much higher expression levels of miR-205-5p than seen in typical cholangiocyte cell lines and healthy controls. Inhibition of miR-205-5p suppressed CCA cell motility, invasion and proliferation of KKU-213B whereby overexpression of miR-205-5p promoted cell proliferation and motility of KKU-100 cells. Bioinformatics tools (miRDB, TargetScan, miRWalk, and GEPIA) all predicted various miR-205-5p targets. Experiments using miR-205-5p inhibitor and mimic indicated that homeodomain-interacting protein kinase 3 (HIPK3) was a potential direct target of miR-205-5p. Overexpression of HIPK3 using HIPK3 plasmid cloning DNA suppressed migration and proliferation of KKU-100 cells. Notably, HIPK3 expression was lower in human CCA tissues than in normal adjacent tissues. High HIPK3 expression was significantly associated with longer survival time of CCA patients. Multivariate regression analysis indicated tissue HIPK3 levels as an independent prognostic factor for CCA patients. These findings indicate that overexpression of miR-205-5p promotes CCA cells proliferation and migration partly via HIPK3-dependent way. Therefore, targeting miR-205-5p may be a potential treatment approach for CCA.

## Introduction

Cholangiocarcinoma (CCA), which constitutes 3% of all gastrointestinal malignancies and 15% of all primary liver tumors, is the second most common primary hepatic malignancy after hepatocellular carcinoma (HCC)^1^. The frequency of CCA varies greatly around the world, with Asia (especially Northeast Thailand) having the highest incidence of the disease and rising fatality rates over the last few decades^2,3^. Northeast Thailand also has a high frequency of liver-fluke (*Opisthorchis viverrini*) infection. This fluke is a known risk factor for CCA and its prevalence in the region is linked to CCA being one of the most prevalent malignancies in both men and women^4^. The patient prognosis for CCA has not significantly improved in the last ten years, with low 5-year survival rates (7–20%) and high tumor-recurrence rates following resection^1^. The disease is typically only detected once it has already spread. Diagnosis is made by combining non-specific biomarkers in blood and/or biopsy samples and imaging techniques^5^. Treatment options are limited for most CCA patients. Therefore, it is hoped that new therapeutic approaches for CCA will emerge from ongoing research into the molecular pathways that underlie the pathogenicity of the disease.

MicroRNAs (miRNAs/miRs) are endogenous, short non-coding RNAs that participate in a variety of biological signaling pathways associated with the cell cycle, apoptosis, proliferation, the immune response and metabolism. They are also associated with biological processes such as organ development and human diseases such as cancer^6,7^. A mature miRNA directs posttranscriptional repression, the mechanism of which is largely dependent on its degree of base-pairing complementarity with target mRNA, and can trigger argonaute protein-catalyzed endonucleolytic cleavage of the mRNA^8^. Such dysregulation has an effect on the characteristics of cancer, such as maintaining proliferative signaling, avoiding growth inhibitors, resisting cell death, inducing invasion and metastasis, and promoting angiogenesis^9,10^. Expression of particular miRNAs could be used as possible biomarkers for clinical diagnosis and prognosis in CCA^11^.

Among the miRNAs implicated in cancer, miR-205 is critical for epithelial physiology because it regulates several pathways involved in differentiation and morphogenesis. Depending on the specific tumor environment and target genes, miR-205 functions as an oncogene in some cell types, promoting tumor initiation and growth. In other cell types, it suppresses cell growth, invasion, and epithelial-mesenchymal transition (EMT), acting as a tumor suppressor^12^. Previous studies proved that overexpression of miR-205-5p has an oncogenic role in lung cancer^13^, endometrial cancer^14^, liver metastases^15^, and nasopharyngeal cancer^16^ and acts as a tumor suppressor in cervical cancer^17^, gall-bladder cancer^18^, triple-negative breast cancer^19^, renal-cell carcinoma^20^, and gastric cancer^21^.

Although miR-205-5p has diverse effects in different malignancies, it is not known how it affects on CCA. The roles of other miRNAs in CCA have been reported previously: there is upregulation of miR-181b-5p^22^ and miR-23a-3p^23^ in human CCA tissues and of miR-196-5p in CCA cell lines^24^. On the other hand, miRNA-206 is downregulated in CCA cell lines^25^, miR-7-5p in intrahepatic CCA tissue and cell lines^26^, and miR-451 in human tissues and serum^27^ in CCA patients compared with normal subjects. As the most upregulated miRNA of the ten miRNAs previously reported to be elevated, miR-205-5p was more abundant in exosomes isolated from KKU-M213 and HuCCA-1 cell lines than in exosomes isolated from the H69 CCA cell line^28^. Hence, the functional analysis of miR-205-5p and the identification of its targets are of interest to us for possible development of CCA therapies.

In the current study, we sought to investigate the relationship between miR-205-5p and its target gene in CCA cells to gain a new perspective on possible molecular targets for CCA treatment. We measured the expression of miR-205-5p in a normal human cell line and three CCA cell lines and human CCA serum samples. We demonstrated the regulatory role of miR-205-5p expression in the proliferation, migration, and invasion of CCA cells. The bioinformatics tools and functional studies suggested that miR-205-5p promoted CCA progression partly through suppressing HIPK3 expression. Our findings help to clarify the regulatory role of miR-205-5p in the development and progression of CCA and identify novel therapeutic target for CCA.

## Results

### Overexpression of miR-205-5p in CCA cell lines and human CCA serum samples

**Figure 1:**
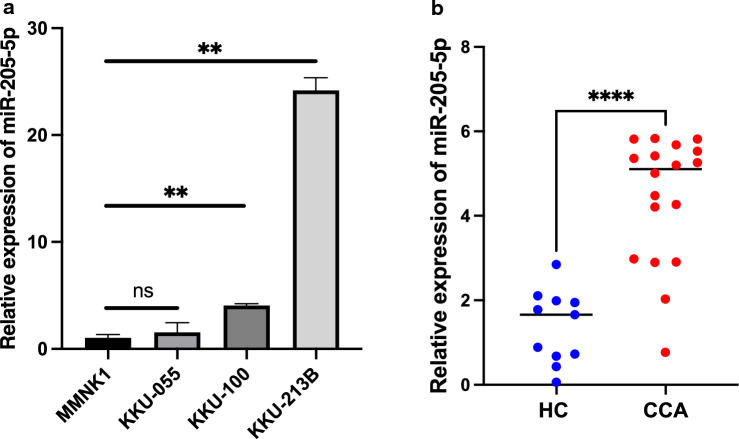
Overexpression of miR-205-5p in CCA cell lines and CCA patients. (**a**) The relative basal expression of miR-205-5p in CCA cells (KKU-055, KKU-100, and KKU-213b), normal cholangiocyte cell lines (MMNK1), and (**b**) in CCA patient serum samples (n = 18) compared with healthy controls (HC, n = 11). Three independent experiments (different cell passages) were each performed in triplicate and expressed as mean ± SD: **p < 0.01, ****p < 0.0001, ns = non-significant.

miR-205-5p was selected as an intriguing miRNA in CCA in our study because it controls actions that are upregulated or downregulated in various epithelial malignancies^12^ and it scored highest among the top-ten upregulated miRNAs that were isolated from exosomes of CCA cells in a previous study^28^. We first examined the expression of miR-205-5p in three CCA cell lines (KKU-213B, KKU-100, and KKU-055) and an immortalized cholangiocyte cell line (MMNK1) by the RT-qPCR. This result showed that the expression of miR-205-5p was higher in all three CCA cell lines than in MMNK1 cells, with the KKU-213B CCA cell line showing the highest overexpression (Fig. 1a). All CCA cell lines showed an overall upregulation of miR-205-5p. Furthermore, the levels of miR-205-5p in serum of CCA patients were significantly higher than that of healthy control subjects (*p* < 0.0001; Fig. 1b). Therefore, we hypothesized that miR-205-5p acts as an oncogene in these cases, and we used subsequent experiments to clarify the function of this microRNA.

### Inhibition of miR-205-5p suppresses cell proliferation, migration, and invasion of CCA cells

**Figure 2:**
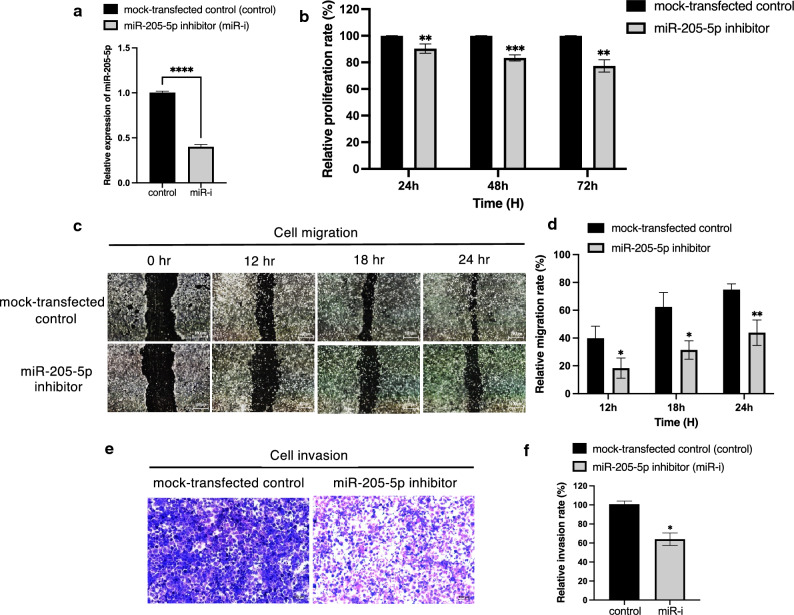
miR-205-5p knocking down affects KKU-213B CCA cell proliferation, migration, and invasion in vitro. (**a**) Relative expression of miR-205-5p to miR-16 as the internal control in the KKU-213B CCA cell line at 48 h after transfection with a miR-205-5p inhibitor, (**b**) Relative proliferation rates of KKU-213B cells transfected with miR-205-5p inhibitor compared to the mock-transfected controls at 24, 48, and 72 h. (**c, d**) The relative cell migration rates of KKU-213B cells transfected with miR-205-5p inhibitor at 12, 18, and 24 h and (**e, f**) invasion rates at 48 h compared to mock-transfected controls. The experiments were performed in triplicate and expressed as mean ± SD (*p < 0.05, **p < 0.01, ***p < 0.001).

The KKU-213B cell line, which had relatively high miR-205-5p expression, was chosen for miR-205-5p knockdown to assess the loss of function of this microRNA in the advancement of CCA cells. The RT-qPCR results confirmed that miR-205-5p expression in this cell line was dramatically reduced after cells were transfected with miR-205-5p inhibitor (Fig. 2a). CCA cell proliferation was measured using the MTT cell proliferation assay at 24, 48, and 72 h after transfecting with the inhibitor. The proliferation rates were significantly lower in the cells transfected with miR-205-5p inhibitor than in the mock-transfected control at each time point (Fig. 2b).

To further our understanding of how miR-205-5p affects the invasive and migratory abilities of human CCA cells, the wound-healing assay and the transwell invasion assay were conducted using the KKU-213B cell line. In the first of these assays, the wound area remained substantially wider (Fig. 2c), and the relative migration rates were significantly lower (Fig. 2d) in the cells transfected with the miR-205-5p inhibitor than in the mock-transfected controls at each time point (12, 18, and 24 h). In the wound-healing assay, cell migration was lower in KKU-213B CCA cells transfected with the miR-205-5p inhibitor.

After 48 h in a cell-invasion (transwell invasion) assay, the invasion of cells, as shown in photos, was remarkably lower (Fig. 2e) and the relative invasion rates were significantly lower (Fig. 2f) in transfected KKU-213B cells than in the mock-transfected controls. In triplicate experiments using different KKU-213B cell passages, the results of cell proliferation, migration, and invasion assays were consistent. The outcomes of these assays indicate that miR-205-5p may operate as an oncogene in CCA.

### Overexpression of miR-205-5p promotes cell proliferation and migration of KKU-100 CCA cell

**Figure 3:**
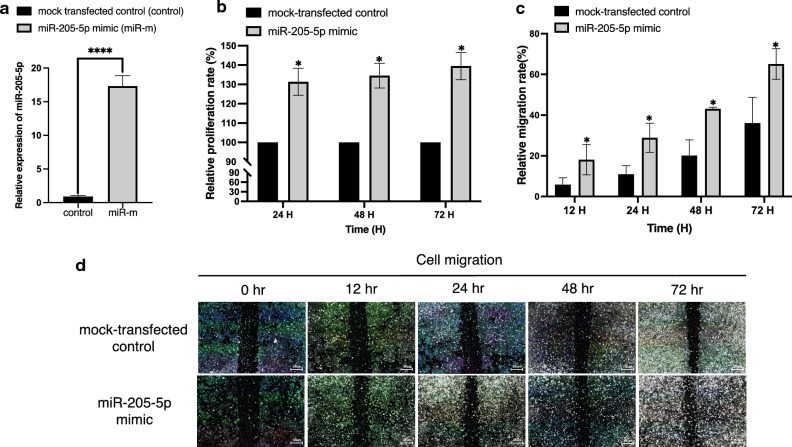
Overexpression of miR-205-5p promotes cell proliferation and migration of KKU-100 cells in vitro. (**a**) Relative expression of miR-205-5p to miR-16 as the internal control in the KKU-100 CCA cell line at 48 h after transfection with an miR-205-5p mimic. (**b**) MTT assay to measure relative proliferation rates of KKU-100 cells transfected with miR-205-5p mimic compared to the mock-transfected controls at all time points (24, 48, and 72 h). (**c, d**) The relative cell migration rates of KKU-100 cells transfected with miR-205-5p mimic compared to mock-transfected controls at 12, 24, 48, and 72 h. Experiments were performed in triplicate and expressed as mean ± SD (*p < 0.05).

To evaluate the gain of function of this microRNA in CCA cells, the KKU-100 CCA cell line, which exhibited low miR-205-5p expression, was transfected with miR-205-5p mimic to promote miR-205-5p overexpression. The RT-qPCR results confirmed that transfection of the cells with a miR-205-5p mimic significantly increased the expression of miR-205-5p compared to mock-transfected control (*p* < 0.0001; Fig. 3a). KKU-100 cells transfected with miR-205-5p mimic had significantly higher proliferation rates than control cells (*p* =  < 0.05; Fig. 3b).

Next, we performed wound healing assay to better understand how miR-205-5p affects the migratory ability of human CCA cells. The result showed that cell migration was significantly increased in KKU-100 cells transfected with the miR-205-5p mimic compared to mock-transfected cells (*p* < 0.05; Fig. 3c and d). According to the results of gain-of-function and loss-of-function experiments, miR-205-5p might exhibit oncogenic role in CCA.

### Expression levels of five predicted target genes of miR-205-5p after transfection with miR-205-5p inhibitor

**Figure 4:**
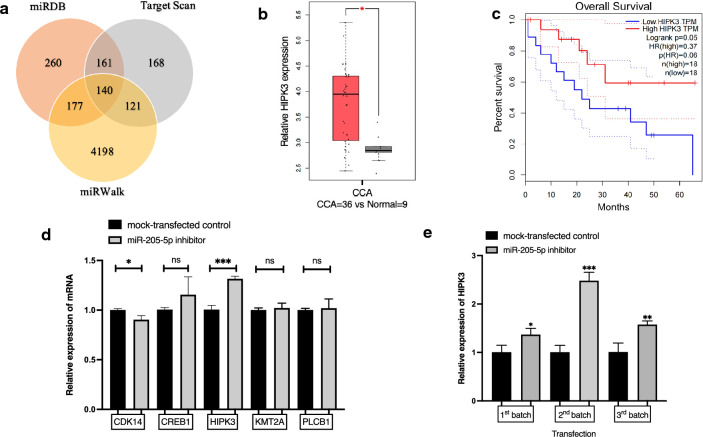
Target prediction of miR-205-5p identifies HIPK3 as one of the potential targets of miR-205-5p. (**a**) The miRDB database predicted 736 potential target genes. For the TargetScan and miRWalk databases, the corresponding numbers were 590 and 4636, respectively. In total, 140 potential target genes were identified in common by all three databases. (**b**) Analysis using existing data from the GEPIA database reveals that the relative expression level of HIPK3 in CCA tissues (n = 36) is higher than in corresponding non-tumorous tissues (n = 9). (**c**) Kaplan–Meier overall survival curves according to the levels of HIPK3 expression. (**d**) Expression levels of five predicted target genes (CDK14, CREB1, HIPK3, KMT2A, and PLCB1) in the miR-205-5p inhibitor-transfected KKU-213B cell line. (**e**) Relative expression levels of HIPK3 in three different batches of KKU-213B cells transfected with miR-205-5p inhibitor. Experiments were performed in triplicate on each batch and expressed as the mean ± SD (*p < 0.05, **p < 0.01, ***p < 0.001).

A bioinformatics study was carried out to identify possible target genes of miR-205-5p. To this end, the databases miRDB, TargetScan, and miRWalk were examined. As shown in Fig. 4a, miR-205-5p had 140 potential target genes in common across the three bioinformatics tools (Supplementary Table S1). Among these, the GEPIA database yielded eight target genes associated with significant overall patient survival log rank p-values, as shown in Supplementary Table. S2. Then, we investigated the relative expression levels of eight significant targets associated with CCA according to the GEPIA database (Supplementary Fig. S1). Finally, we selected five target genes that were significant in both relative expression and log rank p-values (Supplementary Figs. S1 and S2): CDK14, CREB1, HIPK3, KMT2A, and PLCB1 (CDK14=cyclin-dependent kinase 14, CREB1=CAMP responsive element binding protein 1, KMT2A = Histone-lysine N-methyltransferase 2A, PLCB1=Phosphatidylinositol-4,5-bisphosphate phospholipase beta-1). HIPK3 is significantly associated with CCA according to the GEPIA database (Fig. 4b) and appears to act as a tumor suppressor according to overall patient survival data (Fig. 4c). To identify the direct target of miR-205-5p, the expression levels of these five genes were determined using RT-qPCR in KKU-213B cells transfected with miR-205-5p inhibitor. Expression levels of all genes except CDK14 were higher in the transfected cells than in the mock-transfected controls**,** and HIPK3 levels were highest of all (Fig. 4d). The second and third batches of transfection were done on independent occasions using the same procedure to measure the expression in separate biological samples. Figure 4e shows high, substantial, and consistent expression of HIPK3 on all three occasions. This indicates that HIPK3 is a direct target of miR-205-5p.

### HIPK3 might be the direct target gene of miR-205-5p in CCA

**Figure 5:**
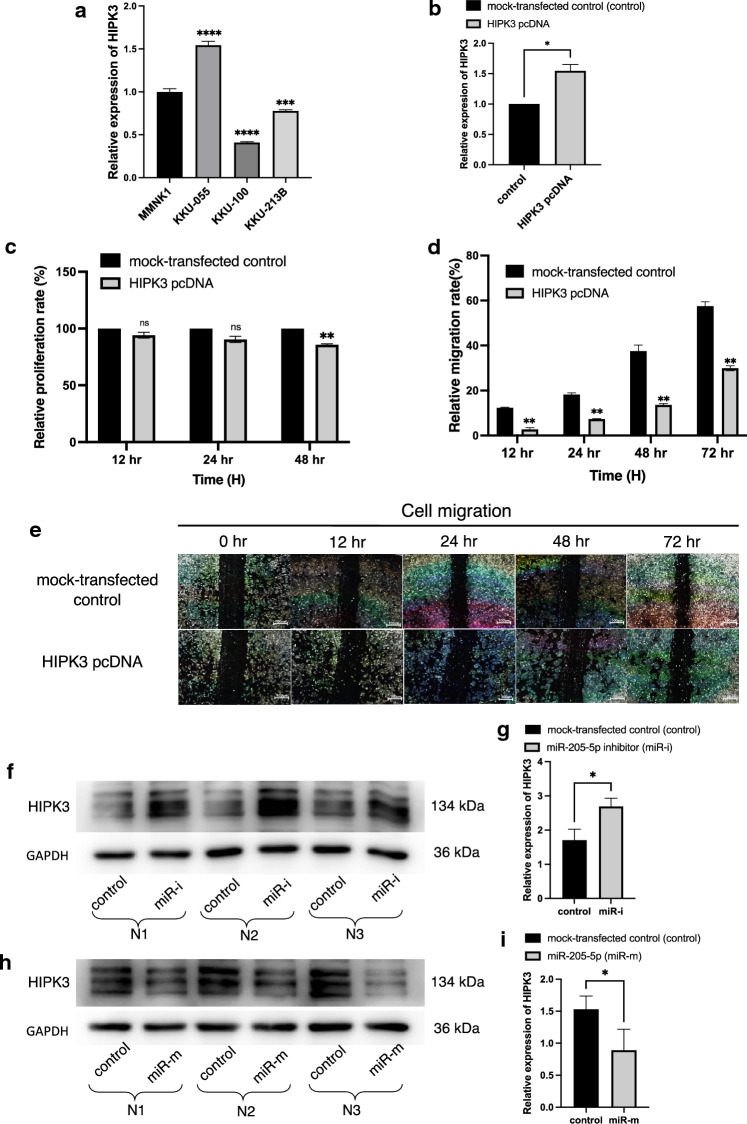
HIPK3 might be direct target of miR-205-5p. (**a**) Relative basal expression of HIPK3 in CCA cells (KKU-055, KKU-100, and KKU-213b) and immortalized cholangiocyte cell line (MMNK1) was measured by RT-qPCR. (**b**) Relative expression of HIPK3 in the KKU-100 CCA cell line was measured at 48 h after transfection with a HIPK3 pcDNA. (**c**) Relative proliferation rates of KKU-100 after transfected with HIPK3 pcDNA were measured using MTT assay at 24, 48 and 72 h after transfection. (**d, e**) The relative cell migration rates of KKU-100 cells transfected with HIPK3 pcDNA compared with mock-transfected controls at 12, 24, 48, and 72 h were measured using wound-healing assay. (**f, g**) KKU-213B cell line was transfected with miR-205-5p inhibitor and (**h**, **i**) KKU-100 cell line was transfected with miR-205-5p mimic and the expression of HIPK3 was measured by Western blot analysis. Experiments were performed in triplicate for each batch and expressed as mean ± SD (*p < 0.05, **p < 0.01, ***p < 0.001, ****p < 0.0001).

We utilized RT-qPCR to examine the expression of HIPK3 in three CCA cell lines (KKU-213B, KKU-100, and KKU-055) and an immortalized cholangiocyte cell line (MMNK1) prior to transfection to select an appropriate CCA cell line for functional study of HIPK3. The result showed that HIPK3 expression was lower in MMNK1 cells than in all CCA cell lines except KKU-055 (Fig. 5a). We therefore transfected KKU-100 cells with HIPK3 plasmid cloning DNA (pcDNA) to evaluate the role of HIPK3 in CCA. Relative expression of HIPK3 was higher than that of mock-transfected control at 48 h after transfection with a HIPK3 pcDNA (Fig. 5b).

The MTT cell proliferation assay was used to assess CCA cell proliferation at 24, 48, and 72 h after HIPK3 pcDNA transfection. At each time point, proliferation rates were significantly lower especially at 72 h in cells transfected with the HIPK3 pcDNA than in the mock-transfected control (*p *< 0.01; Fig. 5c). Next, the KKU-100 cell line was used to gain further insight into whether HIPK3 affects the migratory capacity of CCA cells. In this experiment, KKU-100 CCA cells transfected with HIPK3 pcDNA significantly reduced cell migration in the wound-healing assay compared to mock-transfected controls (*p* < 0.01; Fig. 5d and e). The results of these experiments suggest that the HIPK3 may exhibit tumor suppressor role in CCA.

To confirm whether HIPK3 is a target for miR-205-5p, we assessed HIPK3 expression at the protein level after manipulating the miR-205-5p expression in CCA cells. Western blotting was used to examine HIPK3 protein expression in KKU-213B CCA cells transfected with miR-205-5p inhibitor, and the KKU-100 CCA cell line transfected with miR-205-5p mimic. In comparison with mock-transfected controls, the result showed that the translational levels of HIPK3 were significantly increased in KKU-213B CCA cells transfected with specific miR inhibitor (*p* < 0.05; Fig. 5f and g). On the other hand, the levels of HIPK3 protein were significantly decreased in KKU-100 CCA cell line transfected with specific miR-205-5p mimic (*p* < 0.05; Fig. 5h and i). According to the Western blot results, HIPK3 might be a direct target of miR-205-5p.

**Figure 6:**
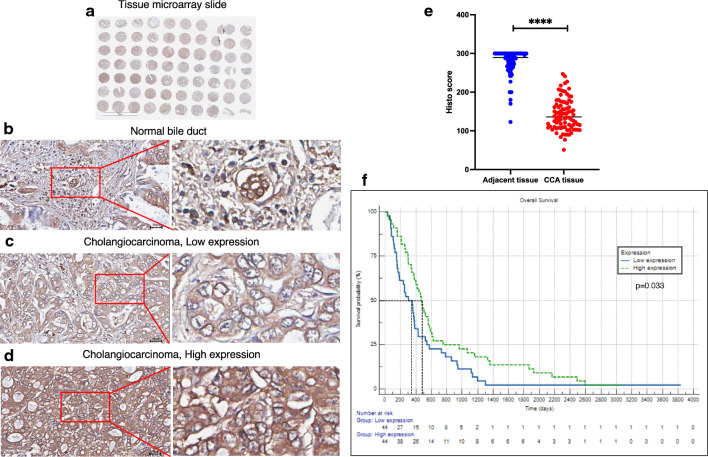
HIPK3 immunohistochemical staining in CCA tissues (magnification, × 40). Immunohistochemistry was used to compare the H-scores of HIPK3 in normal and CCA tissues. (**a**) One entire tissue microarray slide. (**b**) The normal bile duct (NBD) expressed HIPK3 significantly more than the CCA cells. The CCA cells stained weakly positive (**c**) or strongly positive (**d**) for HIPK3 according to H-scores. (**e**) HIPK3 H-scores were compared between healthy and malignant tissues using the Mann-Whitney U test. The median H-score cutoff was 137. High expression was higher than the median (n = 44), and low expression was lower than the median (n = 44). (**f**) The overall survival time of patients with different HIPK3 expression levels (H-score) is shown by Kaplan–Meier curve with number of risk. The experiments were expressed as mean ± SD (****p < 0.0001).

We also used an immunohistochemical (IHC) technique to stain the tissue microarray slides (Fig. 6a) on which were arranged 88 samples from CCA patients. The HIPK3 protein is expressed both intracellularly and on the membrane. Staining intensity was measured, calculated using the H-score formula, and assessed using Image J. Both normal adjacent and CCA tissue showed a positive response to HIPK3 in IHC; however, the degree of the staining was higher in normal adjacent tissue (Fig. 6b) than in low-expression (Fig. 6c) and high-expression (Fig. 6d) CCA tissue**.** Mann–Whitney U test was used to compare H-scores between normal and cancerous tissues of HIPK3 (median H-score = 137). The H-score of HIPK3 in cancerous tissues was significantly lower than that found normal tissues (Fig. 6e)**.** HIPK3 appears to act as a tumor suppressor and could be a direct target of miR-205-5p.

### Correlation between the intensity of HIPK3 and clinicopathologic parameters in CCA

To examine the clinical importance of HIPK3, the median H-score value 137 of 88 cancerous tissues was used as the cutoff to separate low and high HIPK3-expression groups and these were analyzed using Chi-square and Fisher’s exact test (Table 1). We examined whether there was a significant correlation between the HIPK3 expression H-score and clinicopathologic characteristics (gender, age, lymph-node invasion, tumor stage, histological grading, and survival time in days). Apart from the survival-time data, there was no association between HIPK3 expression and the clinical features of CCA patients. We found that the difference in survival days was significant (*p* = 0.033). High HIPK3 scores were associated with longer patient survival in CCA, while low HIPK3 scores were associated with shorter patient survival (Fig. 6f).

**Table 1:** Association between the clinicopathological data of CCA patients and HIPK3 expression levels.

Categories	Number of patients	HIPK3 expression H-score		*p* value
		Low expression≦137	High expression > 137	
Gender				
Female	29	12 (13.6%)	17 (19.3%)	0.257
Male	59	32 (36.4%)	27 (30.7%)	
Age(years)				
≦56	45	23 (26.1%)	22 (25.0%)	0.831
> 56	43	21 (23.9%)	22 (25.0%)	
Lymph node metastasis				
No	48	25 (28.4%)	23 (26.1%)	0.669
Yes	40	19 (21.6%)	21 (23.9%)	
Tumor stage				
I-III	44	23 (26.1%)	21 (23.9%)	0.670
IVA-IVB	44	21 (23.9%)	23 (26.1%)	
Histopathological grading				
Non papillary	44	21 (23.9%)	23 (26.1%)	0.670
Papillary	44	23 (26.1%)	21 (23.9%)	
Survival days				
≦380	44	27 (30.7%)	17 (19.3%)	0.033*
> 380	44	17 (19.3%)	27 (30.7%)	

Clinicopathological data and HIPK3 expression of tissues were analyzed using Chi-square and Fisher’s exact test. Bold values * Statistical significance (*p* < 0.05).

As a result, we also used univariate and multivariate regression analysis to examine the association between survival time (days) and clinicopathological characteristics of CCA patients (Table 2). For each of the variables, the first one listed was assigned to be the reference for all clinicopathological factors in the analysis. Significantly associated with longer survival time were being male, lacking lymph-node metastasis, having papillary tumor type and, in particular, high HIPK3 levels. Most of these associations were significant in both univariate and multivariate analyses. We can postulate that HIPK3 is a prognostic marker for CCA based on the significant correlation between long survival time and high HIPK3 expression.

**Table 2:** Univariate and multivariate regression analysis of the associations between survival time and clinicopathological features of CCA patients.

Variables	Univariate analysis			Multivariate analysis		
	SB	95% CI	*p* value	SB	95% CI	*p* value
Gender						
Female	1			1		
Male	1.128	0.721–1.765	0.599	1.618	1.007–2.600	0.047*
Age						
≦56	1			1		
> 56	0.819	0.535–1.255	0.359	0.690	0.440–1.081	0.105
Lymph node metastasis						
No	1			1		
Yes	0.469	0.300–0.733	0.001*	0.436	0.260–0.730	0.002*
Tumor stage						
I-III	1			1		
IVA-IVB	0.676	0.439–1.040	0.075	1.109	0.663–1.855	0.693
Histopathological grading						
Non papillary	1			1		
Papillary	1.834	1.187–2.832	0.006*	2.660	1.627–4.348	< 0.001*
HIPK3 expression H-score						
Low expression ≦137	1			1		
High expression > 137	1.575	1.024–2.421	0.039*	2.396	1.464–3.922	0.001*

*SB* Survival benefit; *CI* Confidence interval; Bold values * Statistical significance (*p* < 0.05).

## Discussion

CCA is an uncommon cancer, but during the past few decades, its incidence and fatality rates have been rising globally. miRNAs are promising markers for diagnosis and an aid to prognosis in cancers. Such miRNAs and their associated target genes may serve as new therapeutic targets, indicators, or diagnostic and prognostic biomarkers for CCA. However, the function of miR-205-5p and its associated targets in the development of CCA has been uncertain. miR-191 overexpression increases CCA cell proliferation, invasion, and migration in vitro and in vivo by targeting the TET1 gene^29^. Similarly, over-expression of miR-21 and miR-192-5p regulate an oncogene associated with the development of CCA by inhibiting PDCD4 function^30^ and by increasing the proliferation and decreasing apoptosis of CCA cells via the MEK/ERK pathway^31^.

In the present study, we investigated the role of miR-205-5p in the development of CCA. We found that all CCA cell lines had elevated miR-205-5p expression, with the CCA cell line KKU-213B showing the highest expression. As a result, we hypothesized that miR-205-5p might act as an oncogene in CCA. We conducted MTT, wound healing, and transwell invasion assays to demonstrate the functional roles of miR-205-5p in proliferation, migration, and invasion of CCA cells, especially KKU-213B and KKU-100 CCA cells. Our results demonstrated that miR-205-5p exhibits oncogenic potential in CCA.

Each miRNA might influence several target genes in various cancers. To identify potential targets of miR-205-5p in CCA, we used the prediction tools miRDB, TargetScan, miRWalk, and GEPIA. Five potential target genes (CDK14, CREB1, HIPK3, KMT2A, and PLCB1) were eventually chosen for subsequent investigation. Expression levels of these in the CCA cell line after transfection with an miR-205-5p inhibitor were measured. Only HIPK3 expression showed significant upregulation, as verified in three independent experiments. PRKCE, ZEB2, and FAM84B have been explored as targets of miR-205-5p in gallbladder^18^, lung^13^, and gastric cancer^21^, respectively, in previous reports. We hypothesized that the direct target of miR-205-5p would be HIPK3 in CCA.

The homeodomain-interacting protein kinases (HIPKs), including the Ser/Thr kinases HIPK1, HIPK2, and HIPK3, operate as transcriptional co-activators or co-repressors by interacting with homeobox proteins and other transcription factors. HIPKs regulate a variety of biological processes in response to various external stimuli, including signal transduction, apoptosis, embryonic development, DNA damage response, and cellular proliferation^32^. HIPK3 is a tumor suppressor in breast^33^, lung^34^, colorectal^35^, and renal cell carcinomas^36^ as well as in the advancement of esophageal squamous-cell carcinoma tumors^37^. According to our RT-qPCR and Western blot results, the expression of HIPK3 was negatively and significantly correlated with miR-205-5p activity. Similarly, previous study demonstrated that reduction of HIPK3 due to miR-197-3p targeting promoted biologically malignant cell behaviors in breast cancer^33^. Moreover, overexpression of HIPK3 suppressed cell proliferation and migration in CCA cells, particularly in KKU-100 cells, agreeing with that in colorectal cancer in which overexpression of HIPK3 inhibited cancer cell growth and migration^35^.

We have established that the target of miR-205-5p in CCA is HIPK3. In a subsequent experiment, we stained human CCA tissue sections using immunohistochemistry to confirm this: human tissue samples yield a less biased result than do cancer cell lines. In comparisons of CCA tissue with normal adjacent cholangiocytes, the intensity of HIPK3 staining was dramatically higher in the latter based on H-scores. A previous study noted that low HIPK3 expression indicated a poor prognosis in clear-cell renal-cell carcinoma tissues^36^. Furthermore, using Chi-square and Fisher's exact tests, as well as univariate and multivariate regression analysis, we discovered a significant correlation between longer patient survival time and high HIPK3 H-scores in CCA patients. HIPK3, a target of miR-205-5p, therefore acts as a prognostic indicator for CCA. In addition, miR-205-5p and its target, the HIPK3 gene, may be novel targets for development of new CCA treatments. However, it should be noted that, although we did establish the evidence to confirm that HIPK3 is a direct target for miR-205-5p using miR inhibitor and mimic, a more precise technique such as a dual luciferase assay should be performed to confirm this finding.

## In conclusion

In summary, miR-205-5p expression was upregulated in CCA cell lines but downregulated after transfection with a miRNA-205-5p inhibitor. HIPK3 levels rose in transfected cells inversely proportional to the decrease of miR-205-5p. Transfected cells exhibited reduced cell proliferation, migration, and invasion abilities, indicating that miR-205-p has an oncogenic function in CCA.

## Methods

### Sample collection and ethical statement

Fresh 18 serum samples from patients (from January 2023 to August 2023) with intrahepatic CCA were obtained from the Cholangiocarcinoma Research Institute, Khon Kaen University. For controls, 11 serum samples were collected from healthy individuals who underwent check-up at the Medical Technology and Physical Therapy Office of the Health Service, Faculty of Associated Medical Sciences, Khon Kaen University.

Formalin-fixed paraffin-embedded (FFPE) CCA tissue sections from 88 intrahepatic CCA patients were obtained from the Department of Pathology, Faculty of Medicine, Khon Kaen University. The CCA sections were arranged on seven tissue microarray (TMA) slides, each also including two sections of normal pancreas, gallbladder, and colon as controls. The informed consent of human participants in this study was obtained from all subjects under the previous ethics (HE571283). The protocol of the study was reviewed and approved by the Human Ethics Committee of Khon Kaen University (HE662104) for serum and (HE641552) for tissue. The study was conducted according to the guidelines of the Declaration of Helsinki and the ICH Good Clinical Practice Guidelines and approved by the Khon Kaen University Ethics Committee.

### Cell culture

The immortalized cholangiocyte cell line (MMNK1) and three human CCA cell lines (KKU-213B, KKU-100, and KKU-055) were obtained from the Japanese Collection of Research Bio-Resources (JCRB) Cell Bank, Japan. All the cells were maintained in DMEM (Dulbecco's Modified Eagle Medium) with high glucose (4.5 g/L) (Gibco, Thermo Fisher, USA) containing 10% fetal bovine serum and 1% penicillin–streptomycin and then kept in an incubator with a 5% CO_2_ environment at 37 °C. Frozen cell pellets were taken from all cell lines when cell confluency was between 50% and 70% to observe the expression levels of designated miRNAs and expected target genes.

### RNA isolation and reverse transcription

Total RNA was extracted from cell pellets using Thermo Fisher Scientific's PureLinkTM RNA Mini Kit (Carlsbad, USA). Complementary DNA (cDNA) was generated using the Revert Aid First Strand cDNA Synthesis reverse transcription kit (Thermo Fisher Scientific, Carlsbad, USA) for target genes and the qScriptTM microRNA cDNA Synthesis kit for miRNA according to the instructions with each kit. The quality and quantity of total RNA and cDNA were measured using a Nanodrop spectrophotometer (Thermo Fisher Scientific, USA) and gel electrophoresis for target genes.

300 μL of each serum sample was used for the extraction of total RNA, especially miRNA. Total RNA was extracted for serum samples using the NucleoSpin miRNA plasma kit (cat. No. 740981.50, Germany). Complementary DNA (cDNA) was generated using the Agilent miRNA 1stStrand Synthesis (cat. No. STR_600036, USA) for miRNA according to the instructions with each kit. Total RNA and cDNA were measured using a Nanodrop spectrophotometer (Thermo Fisher Scientific).

### Quantitative real-time polymerase chain reaction (RT-qPCR)

An analysis of miRNAs was performed in triplicate using the miScript SYBR Green PCR Kit (Qiagen, Germany) according to the manufacturer's procedures. The thermal cycling parameters were an initial pre-incubation at 95 °C for 15 min, followed by 45 amplification cycles at 94 °C for 15 s, specific annealing temperature for internal control and candidate miRNAs for 30 s, and then 70 °C for 30 s. miR-16 (cat. No. HO00023219) (Macrogen, Thailand) was utilized as an internal control for normalization of the candidate miR-205-5p (cat. No. YP00204487) (Qiagen). A proprietary universal reverse primer (Qiagen) was utilized for both miR-205-5p and miR-16.

For target genes, RT-q PCR was performed in triplicate with the SYBR Green PCR Kit (Thermo Fisher Scientific, Invitrogen) as follows: one pre-incubation step of 95 °C for 15 min, followed by 45 amplification cycles of 95 °C for 10 s, the specific annealing temperature for each gene for 20 s, and 72 °C for 30 s using the Light Cycler 480 II Real-Time PCR System (Roche Diagnostics Ltd., Rotkreuz, Switzerland). GAPDH (Macrogen) was used as the reference gene for the normalization of five predicted target genes; CDK14, CREB1, HIPK3, KMT2A and PLCB1 (Macrogen). The 2^−ΔΔCt^ method was used to quantify the RT-qPCR levels of target genes and miRNAs. The sequences of primers used for RT-qPCR analysis are shown in Supplementary Table S3**.** Every experiment was performed three times.

### Cell transfection

Cell transfection was performed in the KKU-213B CCA cell line using the miR-205-5p inhibitor (cat. No. 4464084, mirVana® miRNA inhibitor, Invitrogen, Thermo Fisher Scientific, USA) for loss of function and in the KKU-100 CCA cell line using miR-205-5p mimic (cat. No. 4464066, mirVana® miRNA mimic, Invitrogen, Thermo Fisher Scientific, USA) for gain of function. Transfection was performed using Lipofectamine™ RNAiMAX Transfection Reagent (cat. No. 13778030, Invitrogen, Carlsbad, Lithuania, USA) in accordance with manufacturer’s instruction. For HIPK3 overexpression, KKU-100 CCA cell line was transfected with HIPK3 pcDNA (Dharmacon cDNA, Horizon Discovery, UK) using Lipofectamine 2000 Transfection Reagent (cat. No. 2471418, Invitrogen, Carlsbad, Lithuania, USA) as per manufacturer’s protocol. The transfected cells were harvested 48 h after the transfection for further experiments.

#### Cell proliferation

The effect of the miR-205-5p inhibitor and mimic on CCA cell proliferation was assessed using the MTT assay. Transfected cells (1 × 10^3^ cells/well) and mock-transfected controls were cultured in 96-well plates for 24, 48, and 72 h in an incubator at 37 °C and a 5 percent CO_2_ atmosphere. After incubation, the old culture medium was replaced with 100 μL of DMEM without fetal bovine serum (FBS) and the addition of 10 μL per well of 5 mg/ml of 3-(4,5-dimethylthiazol-2-yl)-2,5-diphenyl tetrazolium bromide (MTT, Thermo Fisher Scientific) stock in phosphate-buffered saline. Incubation was continued for an additional 4 h in a 37 °C incubator. To each well was added 100 µL of dimethyl sulfoxide (DMSO) to dissolve formazan crystals. A microplate photometer (VARIOSKAN LUX, Thermo Scientific) was then used to measure absorbance at 540 nm. The optical densities (ODs) at each time point were obtained and relative proliferation rates (as percentages) were calculated. Each experiment was carried out three times. The final relative proliferation rate (%) was obtained from the mean value of three repeated experiments.

#### Cell migration

A cell wound-healing experiment was used to evaluate cell migration. CCA cells (7.5 × 10^4 ^cells/well) were seeded onto 24-well plates and cultivated to reach 90 percent monolayer confluency for 24 h. The cells were scraped with a 200 µL plastic pipette tip to produce a linear wound. A fresh aliquot (500 µL) of DMEM was used to replace the old medium. The migration rates were measured, and photographic images were taken under a phase contrast microscope (Nikon, Japan) with 4 × magnification at 0, 12, 18, 24, 48 and 72 h intervals until nearly 100% closure. Cell migration was assessed and reported as a migration index, or the initial width of the wound relative to the width at 12, 18, 24, 48 and 72 h. Each experiment was carried out three times. The mean value of three repeated experiments was used to calculate the final relative migration rate (%).

#### Transwell invasion assay

The cells were trypsinized and resuspended in DMEM without serum after transfection. After cell counting, the density was adjusted to 2 × 10^4^ cells/100 µL of the suspension, and 100 µL was added into the upper chamber containing a Matrigel-coated Transwell insert (200 µg/mL). DMEM supplemented with 10% FBS was then added into the lower chamber as a chemoattractant agent, while cell suspensions in the upper chamber were kept FBS-free. After 48 h of incubation at 37 °C, the chamber was removed and preserved with cold acetone for 30 min then stained with 0.2% crystal violet for 15 min. Cells were then gently wiped off the inner surface of the basement membrane of the chamber and washed with phosphate-buffered saline (PBS) to remove the inner layer cells. Finally, six randomly chosen fields of view (at 10 × magnification) were used to examine the stained outer layer of the chamber's basement membrane, which included cells within the pores, under a microscope. Image J was used for counting the cells and calculating the percentage of area (% area). Then, the relative invasion rates were calculated using the mean value (from three replicates) of area based on the percentage.

### Western blotting

Protein (20 µg) was separated by SDS-PAGE, and transferred to a PVDF membrane. The membrane was blocked and incubated with the primary HIPK3 antibody (Proteintech 25107-1-AP) (1:1000 dilution) diluted in 5% bovine serum albumin (BSA) overnight at 4 °C with gentle shaking. After washing, the membrane was incubated with a horseradish peroxidase (HRP)-conjugated secondary antibody. The immunoreactive band was developed and visualized using an UltraScence Western Substrate (Bio-Helix) enhanced chemiluminescence detection kit (ECL Prime, GE Healthcare) and detected using Amersham ImageQuant 800 (GE Healthcare Bio-Sciences AB). Relative band intensity was measured using Image J software.

### Histopathological assessment (immunohistochemical study)

The paraffin-embedded tissue sections in the tissue microarray were deparaffinized in xylene, then rehydrated in decreasing concentrations of ethanol (100%, 95%, and 70%, 3 times each), and finally in water for 5 min. After rehydration, the antigen was retrieved by autoclaving with sodium citrate buffer (10 mM sodium citrate, 0.05% Tween 20, pH 6.0). The slides were immersed in 3% H_2_O_2 _for 10 min to quench endogenous peroxidases. Then, non-specific binding sites were blocked with 5% BSA by incubating the slide for 1 h at room temperature. The sections were then incubated with rabbit anti-HIPK3 polyclonal antibody (1:400 dilution; cat. No. 25107-1-AP, Proteintech, USA) diluted in 5% BSA solution at 4 °C overnight. To establish the specificity of the primary antibody, control sections were treated with just the antibody diluent. On the next day, the slides were washed with PBS and then incubated with a diluted secondary antibody in a 5% BSA solution for 1 h at room temperature. Finally, 3,3-diaminobenzidine (DAB) was added to generate immunoreactivity, and the slides were counterstained with Mayer hematoxylin. Permount™ (Thermo Fisher Scientific) and coverslips were used to mount the slides. The intensity of staining was checked under an ordinary light microscope and calculated using the H-score formula, and assessed using Image J. The immunohistochemistry staining of HIPK3 was examined using the Histo-score (H-score) method, in which the intensity level (0 = none; 1 = faint; 2 = moderate; and 3 = high) and the H-score of stained cells was quantified and ranged from 0 to 300 in each case as follows: HistoScore = (1 × % weakly stained cells) + (2 × % moderately stained cells) + (3 × % strongly stained cells)^38^.

### Bioinformatics analysis

The bioinformatics tools TargetScanHuman7.0 (accessed in November 2020), miRDB (accessed in November 2020) and miRWalk2.0 (accessed in November 2020) were used to predict the targets of miR-205-5p. For validating target identification, GEPIA (Gene Expression Profiling Interactive Analysis) (accessed in January 2021) was also used for confirmation of predicted target genes in CCA for miR-205-5p.

### Statistical analysis

All statistical analyses were performed using GraphPad Prism 9 software (GraphPad Software Inc., San Diego, CA, USA) and SPSS statistics. Values are reported as the mean and standard deviation, and significance was evaluated using a two-tailed Student's paired t-test. Chi-square and Fisher’s Exact test were used to determine the associations between HIPK3 and patients’ clinicopathological data. Parameters were analyzed using univariate and multivariate regression analyses to determine relative correlation with lymph node metastasis, histopathological grading (H-scores) and HIPK3 expression. Kaplan–Meier survival curves were also used for patient survival analysis. A *p* value ≤ 0.05 indicated statistical significance.

### Supplementary Information

Supplementary Information.

## Data Availability

The data that support the findings of this study are available upon reasonable request from the corresponding author.
