# Supplementary material for: Overexpression of microRNA-205-5p promotes cholangiocarcinoma growth by reducing expression of homeodomain-interacting protein kinase 3
Source: Sci Rep. 2023 Dec 17;13:22444. doi: 10.1038/s41598-023-49694-x (PMC10725890; doi:10.1038/s41598-023-49694-x)
Supplement: Supplementary file 1 — Supplementary Information. [file 41598_2023_49694_MOESM1_ESM.pdf]

Overexpression of microRNA 205-5p promotes cholangiocarcinoma growth by reducing expression of homeodomain-interacting protein kinase 3

Supplementary information

Aye Myat Mon[1,9], Kitti Intuyod[2,9], Sirinapha Klungsaeng[3,9], Apinya Jusakul[4,9], Thatsanapong Pongking[5,9], Worachart Lert-itthiporn[6,9], Vor Luvira[7,9], Chawalit Pairojkul[2,9], Tullayakorn Plengsuriyakarn[8], Kesara Na-Bangchang[8], Somchai Pinlaor[3,9], Porntip Pinlaor[4,9*]

[1]*Medical Technology Program, Faculty of Associated Medical Sciences, Khon Kaen University, Khon Kaen 40002, Thailand*

[2]*Department of Pathology, Faculty of Medicine, Khon Kaen University, Khon Kaen 40002, Thailand*

[3]*Department of Parasitology, Faculty of Medicine, Khon Kaen University, Khon Kaen 40002, Thailand*

[4]*Centre for Research and Development of Medical Diagnostic Laboratories, Faculty of Associated Medical Sciences, Khon Kaen University, Khon Kaen 40002, Thailand*

[5]*Biomedical Science Program, Graduate School, Khon Kaen University, Khon Kaen 40002, Thailand*

[6]*Department of Biochemistry, Faculty of Medicine, Khon Kaen University; Khon Kaen 40002, Thailand*

[7]*Department of Surgery, Faculty of Medicine, Khon Kaen University; Khon Kaen 40002, Thailand*

[8]*Graduate Program in Bioclinical Sciences, Chulabhorn International College of Medicine, Thammasat University (Rangsit Campus), Pathumthani, Thailand, Center of Excellence in Pharmacology and Molecular Biology of Malaria and Cholangiocarcinoma, Thammasat University (Rangsit Campus), Pathumthani, Thailand*

[9]*Cholangiocarcinoma Research Institute, Faculty of Medicine, Khon Kaen University, Thailand*

* Corresponding author at: Faculty of Associated Medical Science, Khon Kaen University, KhonKaen, Thailand. Tel.: +66 43 202086, +66 43 348 387; fax: +66 43 202086, +66 43 202 475.

*E-mail address:*porawa@kku.ac.th (P. Pinlaor).

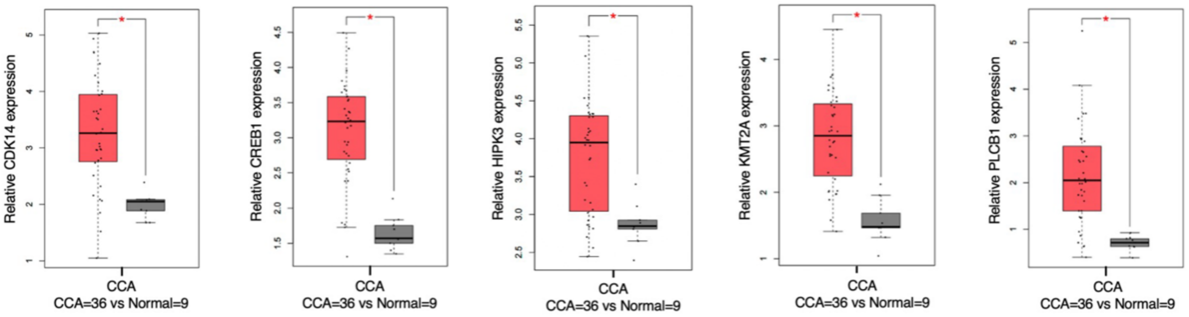

Supplementary Figure S1. Relative expression of five predicted target genes of miR-205-5p in CCA tissues and non-tumorous tissues. Data collected from the GEPIA database showed the relative expression levels of five predicted target genes of miR-205-5p (CDK14, CREB1, HIPK3, KMT2A and PLCB1) in CCA tissues (n=36) and their corresponding non-tumorous tissues (n=9).

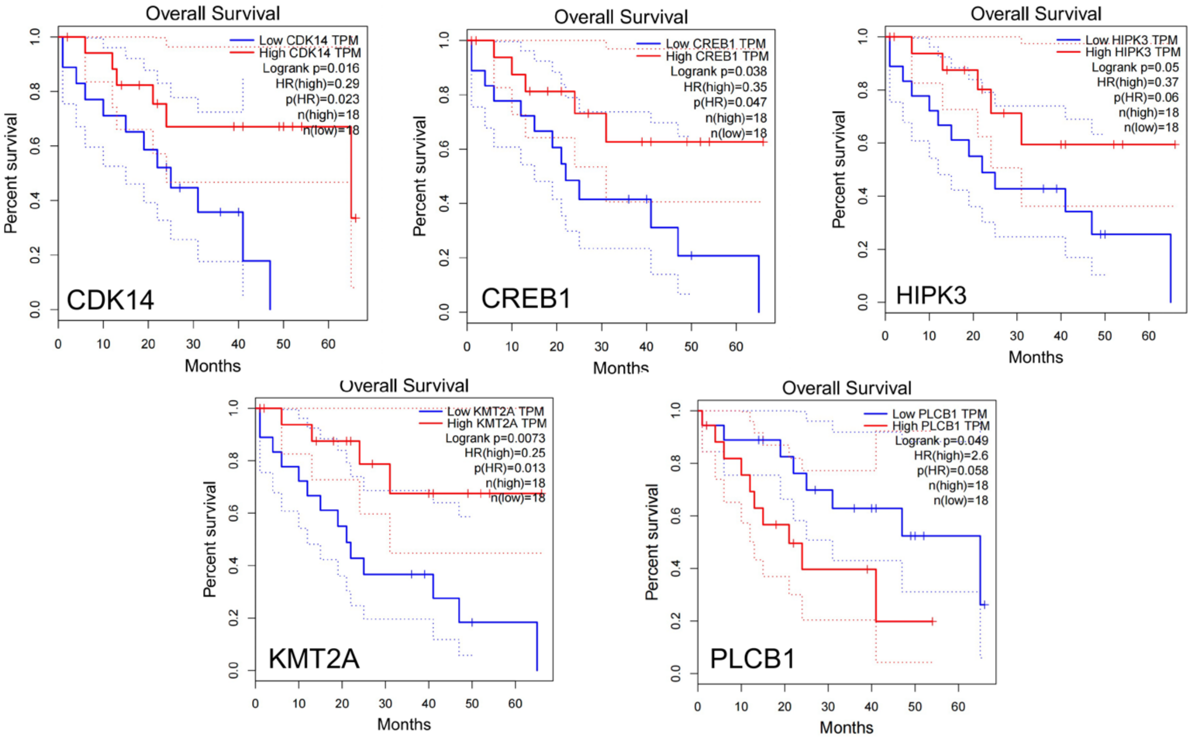

Supplementary Figure S2. Overall survival of five predicted target genes of miR-205-5p CCA patients. Data collected from the GEPIA database showed Kaplan–Meier overall survival curves according to the expression levels of five predicted targets genes of miR-205-5p (CDK14, CREB1, HIPK3, KMT2A and PLCB) in CCA patients (n=36).

Supplementary Table S1. Common 140 target genes and their p-values GEPIA database for miR-205-5p in intersection of three databases miRDB, TargetScan and miRWalk.

No	Common target genes	Log-rank p-value in GEPIA	No	Common target genes	Log-rank p-value in GEPIA	No	Common target genes	Log-rank p-value in GEPIA
1	AAK1	0.58	48	HERC3	0.94	95	RPS6KA3	0.049
2	ABHD10	0.49	49	HIPK3	0.05	96	RTN3	0.54
3	ABHD17B	0.12	50	HOOK3	0.16	97	RUNX2	0.18
4	ABI2	0.37	51	IL6ST	0.22	98	SALL4	0.87
5	ACSL1	0.16	52	JPH4	0.11	99	SATB2	0.23
6	AEBP2	0.86	53	KMT2A	0.0073	100	SBF2	0.65
7	AMOT	0.71	54	LCOR	0.12	101	SCMH1	0.4
8	ARHGRP24	0.54	55	LDLRAD3	0.58	102	SECISBP2L	0.34
9	ATP7A	0.98	56	LHFPL2	0.083	103	SEMA4C	0.37
10	B3GNT5	0.44	57	LIMS2	0.46	104	SERTAD2	0.57
11	BMF	0.7	58	LPAR1	0.86	105	SIAH1	0.31
12	BMPER	0.13	59	LRRK2	0.29	106	SIPA1L1	0.13
13	BTBD3	0.73	60	LYPD6	0.31	107	SLA30A7	0.55
14	CADM1	0.51	61	LYSMD3	0.23	108	SLC4A4	0.53
15	CALCRL	0.69	62	MAGI1	0.33	109	SMIM14	0.12
16	CCDC43	0.95	63	MAGI2	0.26	110	SNX27	0.5
17	CDC27	0.84	64	MDM4	0.98	111	SORBS1	0.22
18	CDH11	0.58	65	MED1	0.2	112	SPRY1	0.31
19	CDH7	0.82	66	MED13L	0.26	113	SRSF10	0.04
20	CDK14	0.016	67	MERN1	0.76	114	STK3	0.13
21	CDK19	0.21	68	MMD	0.81	115	SULF1	0.71
22	CENPF	0.17	69	MPRIP	0.39	116	TBX3	0.84
23	CFL2	0.35	70	MSI2	0.2	117	TM9SF3	0.21
24	CHIC1	0.8	71	MTRF1L	0.91	118	TNFANP8	0.42
25	CMTM4	0.53	72	MYLK4	1	119	TNRC6B	0.44
26	CREB1	0.038	73	NACC2	0.95	120	TP53INP1	0.8
27	CREBRF	0.23	74	NFIA	0.1	121	TSHR	0.8
28	CSF1	0.82	75	NKX2-3	0.086	122	TXNRD1	0.58
29	CTPS2	0.35	76	NTNG1	0.5	123	UNC5D	0.56
30	DMXL1	0.37	77	PAFAH1B1	0.39	124	USP13	0.33
31	DSC2	0.74	78	PARD6B	0.15	125	VASN	0.87
32	ELAVL4	0.66	79	PHB	0.58	126	VEGFA	0.16
33	ELF1	0.17	80	PHC2	0.56	127	WDTC1	0.53
34	ENC1	0.32	81	PHYHIPL	0.07	128	WWC1	0.36
35	ENPP4	0.71	82	PLCB1	0.049	129	WWC2	0.12
36	EPB41	0.21	83	PPP1R15B	1	130	WWC3	0.38
37	EPB41L1	0.98	84	PPP1R1C	0.96	131	YAP1	0.14
38	ERB29	0.87	85	PPP1R8	0.14	132	ZCCHC14	0.13

39	ESRRG	0.52	86	PSD3	0.62	133	ZDHHC9	0.24
40	EVA1C	0.32	87	PTCHD1	0.077	134	ZEB2	0.3
41	FAM104B	0.033	88	PTK7	0.91	135	ZFYVE16	0.73
42	FAM136A	0.6	89	QKI	0.38	136	ZHX3	0.44
43	FBXO22	0.23	90	RAB9B	0.68	137	ZNF518B	0.87
44	FSD1L	0.7	91	PBPMS2	0.23	138	ZNF652	0.3
45	GCC2	0.061	92	RGPD4	0.42	139	ZNF800	0.022
46	GLIS3	0.29	93	RNF157	0.2	140	ZSWIM4	0.63
47	GRAMD1C	0.61	94	RORA	0.52			

Supplement Table S2. Eight significant target genes of miR-205-5p according to GEPIA database

No	Significant predicted targets of miR-205-5p	Log-rank p-values in the GEPIA database
1	CDK14	0.016
2	CREB1	0.038
3	FAM104B	0.033
4	HIPK3	0.05
5	KMT2A	0.0073
6	PLCB1	0.049
7	RPS6KA3	0.049
8	ZNF800	0.022

Supplementary Table S3. Sequences of primers used for qRT-PCR analysis

miRNA/Gene	Forward primers (5'-3')	Reverse primers (5'-3')
miR-205-5p	UCCUUCAUUCCACCGGAGUCUG	Proprietary universal reverse primer
miR-16	UAGCAGCACGUAAAUAUUGGCG	Proprietary universal reverse primer
CDK14	CCAAGGAGTTGCTGCTTTTC	TGCCGACAGTCTGTTCTTTG
CREB1	ACATGGAAAAGGGCAAACTG	CAAATGTCCCCAGAAGAGGA
HIPK3	CTGAGAGTGTGGCTGGTTCA	CCAGACAACATGTGCAATCC
KMT2A	CACAACTGGGGACATCACAG	GGTTCACATGCTGAAGCTGA
PLCB1	CGTGGCTTTCCAAGAAGAAG	GCTTCCGATCTGCTGAAAAC
GAPDH	GTCTCCTGACTTCAACAGCG	ACCACCCTGTTGCTGAGCCAA

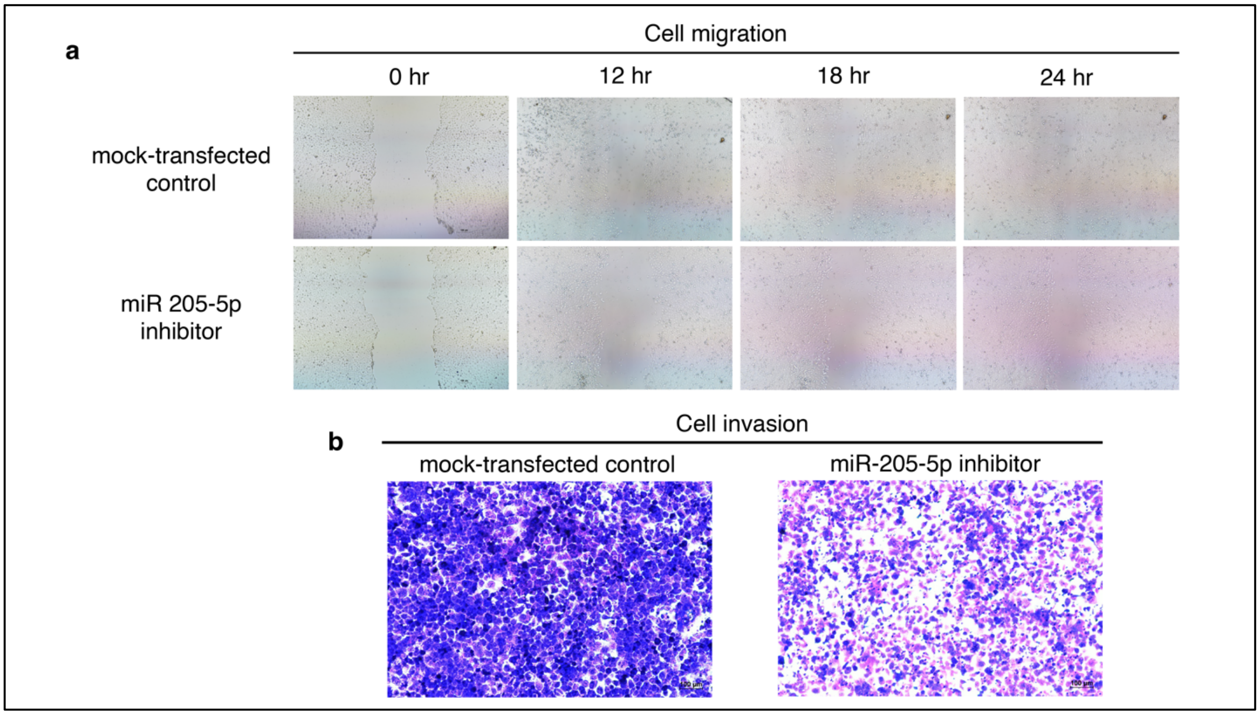

Supplementary Figure S3. High-quality original photographs of (a) cell migration and (b) invasion in KKU 213B CCA cells after transfection with specific miR inhibitor that represent in Fig. 2b and 2d.

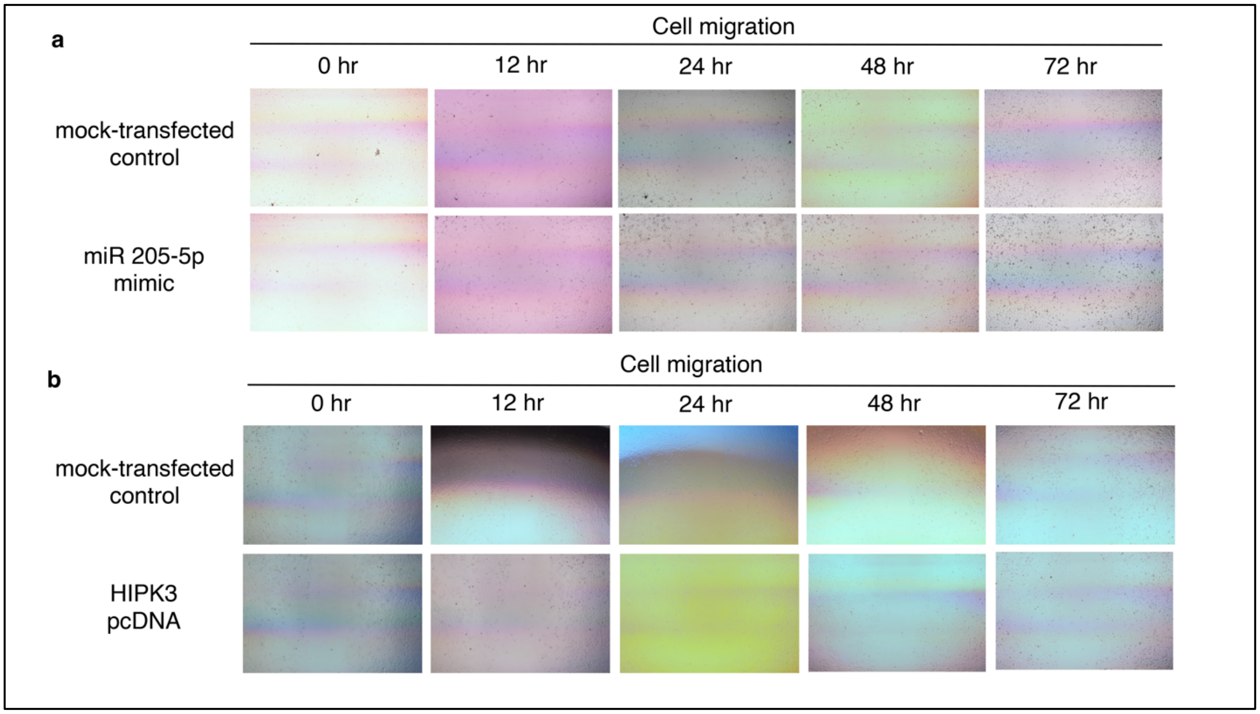

Supplementary Figure S4. High-quality original photographs depicting (a) cell migration in KKU-100 CCA cells following transfection with a specific miR mimic, as shown in Fig. 3b and

(b) cell migration in KKU-100 CCA cell after transfection with HIPK3 pcDNA, represent in Fig. 5d.

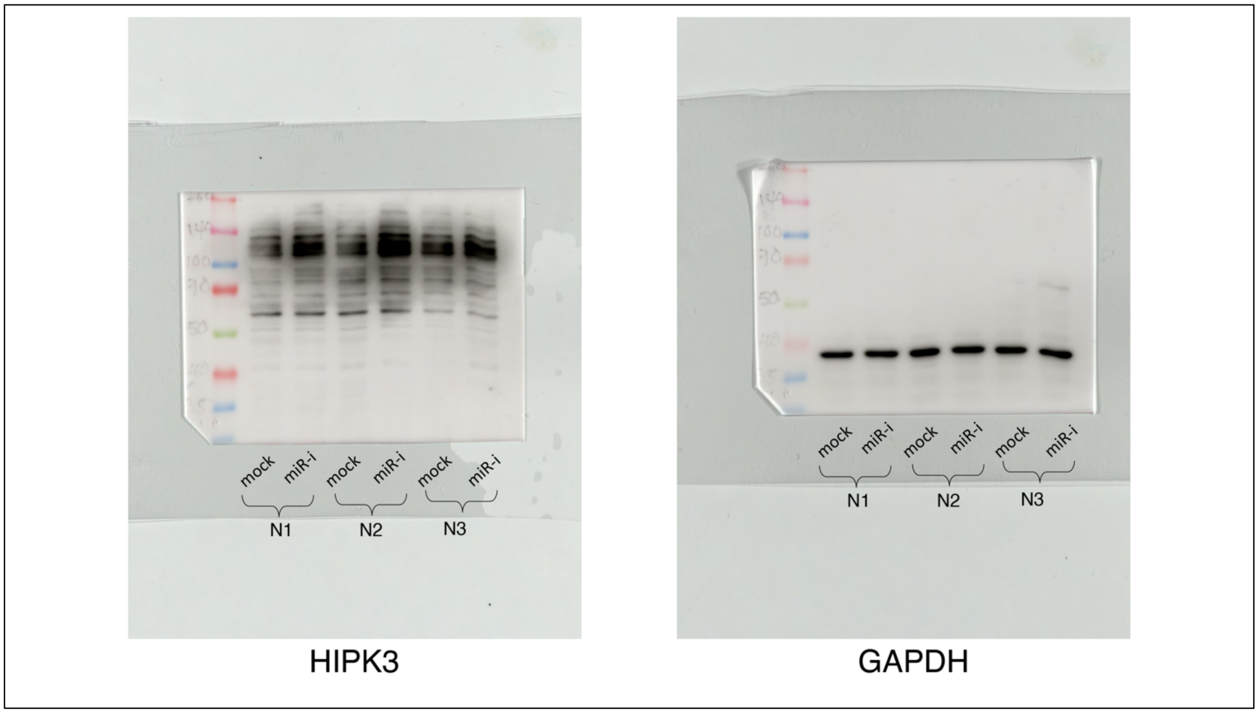

Supplementary Figure S5. Western blot full gel photos of HIPK3 protein and normalized GAPDH control in KKU-213B cell after three transfections (N1, N2, N3) with miR-205-5p inhibitor (Fig. 5f in the manuscript).

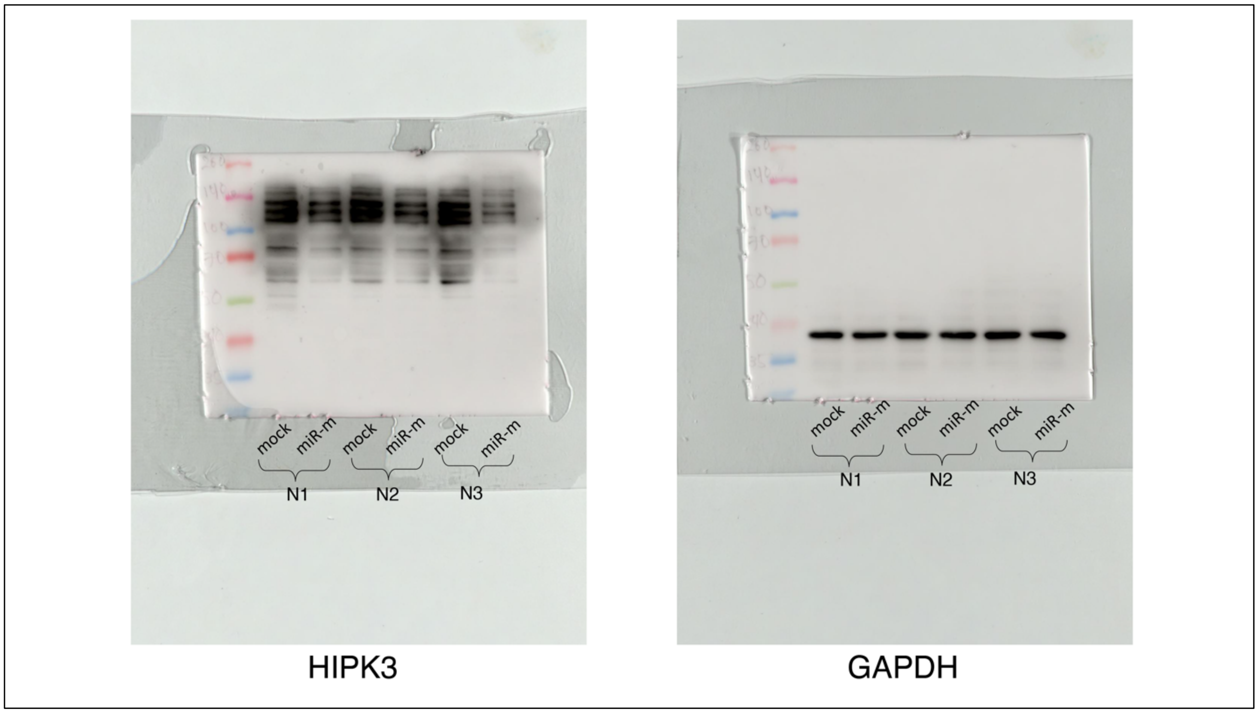

Supplementary Figure S6. Western blot full gel photos of HIPK3 protein and normalized GAPDH control in KKU -100 cell after three transfections (N1, N2, N3) with miR-205-5p mimic (Fig. 5h in the manuscript).
